# Haplotype-resolved genome assembly of ‘Manhattan’ perennial ryegrass (*Lolium perenne* L.) and characterization of drought responsive late embryogenesis abundant genes

**DOI:** 10.1186/s12864-025-12144-1

**Published:** 2025-11-20

**Authors:** Matthew D. Robbins, B. Shaun Bushman, Joseph Gallagher, Peter J. Maughan, Ryan Hayes

**Affiliations:** 1 USDA-ARS Forage and Range Research Laboratory, Logan, UT United States; 2 USDA-ARS Forage Seed and Cereal Research Unit, Corvallis, OR United States; 3 Plant and Wildlife Sciences, Brigham Young University, Provo, UT United States

**Keywords:** Late embryogenesis abundant, LEA, Dehydrin, Grass synteny

## Abstract

**Background:**

Perennial ryegrass is a premier forage and turf grass, a genomic model organism for cool-season perennial grasses, but has relatively poor persistence under drought stress. The cultivar ‘Manhattan’ is an heirloom turf-type cultivar, and ancestral to many current turf cultivars and breeding lines in the USA. To improve turf-type perennial ryegrass genome resources, we assembled and compared two haplotypes of ‘Manhattan’ and extracted late embryogenesis abundant (LEA) gene families.

**Results:**

Both haplotypes resolved into 2.3 Gb genome assemblies of 7 chromosomes each, with Gypsy-like retrotransposon concentrations in putative centromere regions. Repeat content was 83%, and the two haplotypes were syntenic with each other as well as published forage-type perennial ryegrass genomes. Annotations resulted in 43,000 genes for each haplotype with over 95% complete, 89% as single copy genes, and 86% with functional annotation. Seventy-two LEA genes were identified in haplotype-1 and fitted into 8 Pfam-based families, with 46 exhibiting expression evidence in vegetative tissues and 39 showing differential expression upon drought stress.

**Conclusions:**

Broad synteny but high heterozygosity characterized the ‘Manhattan’ perennial ryegrass genome haplotypes. Repeat content, including long terminal repeat genes with annotation support, were high and indicative of cool-season Poaceae grasses. The identification of LEA genes differentially expressed upon drought stress provide candidate genes for further drought tolerance studies.

**Supplementary Information:**

The online version contains supplementary material available at 10.1186/s12864-025-12144-1.

## Background

Perennial ryegrass (*Lolium perenne* L.) is a caespitose, perennial, cool-season grass used as both a forage and turf. Having diploid (2*n* = 2*x* = 14) and auto-tetraploid (2*n* = 4*x* = 28) populations and cultivars, it crosses and produces fertile hybrids with annual ryegrass (*L. multiflorum* Lam.), Italian ryegrass (*L. multiflorum* Lam. ssp. *italicum* (A. Braun) Schinz & R. Keller), and meadow fescue (*Festuca pratensis* Huds.). Diploid perennial ryegrass turf-type cultivars have the highest quality of all cool-season turfgrass species due to their soft leaves and dark green color, and are characterized by relatively rapid germination and establishment [1]. Cultivars of turf-type perennial ryegrasses are used in mixes with Kentucky bluegrass (*Poa pratensis* L.) to provide rapid germination in a seeded lawn, overseeded on hybrid bermudagrass (*Cyanodon dactylon* (L.) Pers.x *C. transylvanensis* Burtt Davy) to provide winter color and function, and planted in disturbed areas to quickly fill in bare soil.

A recent emphasis of perennial ryegrass function under drought stress [2, 3] has increased the demand for drought tolerant varieties. Under drought stress, perennial ryegrass plants exhibited reductions of root/shoot ratios, chlorophyll fluorescence, regrowth after cutting, and plant height [2, 3]. Other studies reported drought-stressed perennial ryegrasses exhibit reduced leaf water content and thinning stand densities [4, 5]. Most of these drought response and gene expression research efforts in perennial ryegrass have focused on wild collections or forage-type cultivars, with limited inclusion of turf-type germplasm.

LEA proteins were initially identified in the desiccation phase of maturing embryos in 1981 [6] from cDNA screens of cotton (*Gossypium hirsutum* L.). Several LEA gene classification methods have emerged, with one main method separating LEAs into eight Pfam-based groups [7] and another into 3–6 groups based on motifs, glycine content, and hydrophilicity [8]. The classification methods produce similar groupings [9], such that LEA genes from newly studied organisms fit equally well into either method. Also called intrinsically disordered proteins (IDPs), LEA genes generally have a lack of structure in solution that can change in some groups, such as Pfam-based LEA_1 genes that form alpha-helices in their N-terminal regions under drought stress and upon substrate binding [10]. Their unstructured confirmation renders LEAs less susceptible to protein denaturation that can occur upon desiccation [11], which allows them to function in protein stabilization, membrane stabilization, and molecular chaperone activities under drought stress [12]. In particular, dehydrin (DHN), LEA_1, and LEA_4 genes have been induced in vegetative drought and desiccation situations [13, 14]. Although sequence homology across groups is minimal, each LEA group has characteristic motifs that are specific to that group [8, 15].

In perennial grasses, LEA genes have been poorly described due their low sequence homology between families and the lack of genomic references. However, in a diverse set of 192 perennial ryegrass plants an evaluation of SNP markers and drought-response traits identified a significant association between a LEA_4/group 3 gene and leaf water content [5]. In a comparison across three cool-season perennial turfgrass species, including perennial ryegrass, all drought-stressed plants showed multiple LEA isoforms induced when compared to unstressed plants [16]. Given the established role of LEA genes in drought response and a need for more drought-tolerant turf cultivars, a characterization of LEA genes in perennial ryegrass is needed to allow for its robust genetic improvement.

Initial genome assemblies for perennial ryegrass were based on Illumina short reads for forage type perennial ryegrass [17] and annual ryegrass [18]. These assemblies produced genomes with the expected haploid chromosome count of seven and an approximate size of 2.2 Gb. Later, sequencing with Oxford Nanopore and PacBio long reads led to full genome assemblies of the forage-type perennial ryegrass cultivar ‘Kyuss’ [19, 20] and a breeding line P226/135/16 (hereafter referred to as P226) [21] and confirmed the genome size and arrangement. No turf-type perennial ryegrass genome assemblies have been sequenced and characterized.

In this paper we provide a haplotype-resolved assembly of the diploid turf-type perennial ryegrass cultivar ‘Manhattan’, an heirloom cultivar in the ancestry of many recent turf varieties. We phase each chromosome with Hi-C, align and orient the chromosomes similar to previous perennial ryegrasses and Triticeae grasses, and show evidence of centromeric and telomeric regions. Annotation methods used long-read CCS sequences, sequences from a drought vs. control RNAseq experiment including ‘Manhattan’, and public EST databases. Using these resources, we show the location of LEA genes in the genome, the homology within LEA gene families, and expression evidence of LEA genes within each family that are highly induced upon drought stress in vegetative tissues.

## Methods

### Genome sequencing and assembly

A single plant from seed of ‘Manhattan’ obtained from the USDA-ARS Forage and Cereal Seed Research Unit in Corvallis, Oregon was used for sequencing and assembly. Flow cytometry for ploidy and genome size estimation used a propidium iodide staining solution (BioSure, Grass Valley, CA) and an Accuri C6-Plus flow cytometer (BD Biosciences, Franklin Lakes, NJ). A previously sequenced Poa annua genome of 1.8 Gb in size [22] was used as a relative reference. DNA was extracted from young leaves of the ‘Manhattan’ plant using a Zymo Genomic DNA extraction kit (Zymo Research, Irvine, CA), and a HiFi long read library was prepared using a SMRTbell template preparation kit v1.0 (PacBio, Menlo Park, CA). PacBio sequencing was conducted on a Sequel II instrument at the Brigham Young University DNA Sequencing Center (Provo, UT, USA).

To obtain estimates of genome size and heterozygosity, PacBio HiFi [23] reads were analyzed using Jellyfish v. 2.2.9 [24] with the -C option and k-mer size of 21, and GenomeScope v. 2.0 [25]. PacBio HiFi and Omni-C reads were assembled using hifiasm v. 0.16.0 [26] using default parameters to produce haplotype-resolved assemblies. Contigs within a haplotype were scaffolded by Dovetail Genomics (Scotts Valley, CA, USA) using proximity ligation with their Omni-C and HiRise pipelines [27, 28].

To assess quality of the scaffold assemblies, (1) assembly metrics were produced using the --large option of QUAST v. 5.2.0 [29]; (2) gene completeness was assessed with BUSCO v5.4.7_cv1 [30] using the poales_odb10 dataset (creation date 8/5/2020); (3) telomeres were identified by dividing the genome into 500 kb windows using the makewindows command in BEDTools v. 2.26.0 [31] and counting occurrences of the forward and reverse complement of the TTTAGGG plant-type tetramer [32] in each window using the BEDTools nuc command with the --pattern option; and (4) the original HiFi reads were mapped back to the assembly using the -x map-hifi preset option of minimap2 v. 2.22 [33]. Scaffold sequences were analyzed using BlobTools2 v. 2.5.0 [34] and Kraken 2 v. 2.1.1 [35] to identify potential contaminants. Data provided to BlobTools2 included blastn (blast + v. 2.11.0 [36]), hits of assembly scaffolds against the nt database, original HiFi read coverage, and BUSCO scores. The PlusPFP-16 RefSeq database [37] was used for Kraken 2. Scaffolds were deemed contaminants and removed if the taxon was assigned outside Streptophyta, the GC percentage was extreme (40% >GC > 50%), or the read coverage was less than five. Scaffolds with greater than 99% query coverage, after BLASTn alignment to the plastid RefSeq database (release 207) [38], were considered chloroplast and removed from the assembly. Due to the complexity of the mitochondrial genome in plants [39–41], scaffolds with the best nt database hit to mitochondrial genome sequence, or with greater than 40% query coverage to the mitochondrial RefSeq database, or scaffolds for which all functional gene annotations were mitochondrial, were considered mitochondrial sequences and removed from the assembly. Repetitive elements in scaffolds were identified using RepeatModeler2 v2.0.2a [42] to create a custom repeat library for RepeatMasker v. 4.1.2-pl [43], which located repeats and masked the assemblies prior to annotation. Scaffolds with less than 10 Kb of unmasked sequence were considered highly repetitive and removed from the assembly. The LTR Assembly Index (LAI) [44] was then calculated with LTRharvest [45] through GenomeTools v1.6.3 [46], LTR_FINDER_parallel v1.1 [47], and LTR_retriever v2.9.6 [48]. Gaps within scaffolds were identified using the gap function of seqtk v1.4-r130-dirty [49]. Chromosome-level scaffolds were aligned with previously reported perennial ryegrass genomes [19, 21] using CHROMEISTER v1.5.a [50] and the seqtk seq utility with the -r flag was used to reorient scaffolds if necessary to match these genomes. Chromosomes were named ChrX_Lpman1 for haplotype 1 or ChrX_Lpman2 for haplotype 2, where X is the chromosome number and Lpman is abbreviation for *Lolium perenne* cultivar ‘Manhattan’.

Chloroplast and mitochondrial genomes were assembled from PacBio HiFi reads using Oatk v1.0 [51] with syncmer size as 1001 and syncmer coverage as 180 using the embryophyta database.

### cDNA sequencing and genome annotation

Both Iso-Seq, under various treatments, and RNA-seq, specifically under drought treatments, data were collected for evidence to annotate each haplotype separately. For Iso-Seq, total RNA was collected from leaf, crown, and inflorescence tissues under standard greenhouse, salt stress, and cold stress treatments for general gene expression. Salt treated plants were irrigated with deionized water and 50 mM NaCl for three days prior to tissue collection, and cold stressed plants were transferred to a growth chamber at 4℃ and 8 h light (500 µmol m^−2^ s^−1^) for three days prior to tissue collections. The total RNA from these tissues and treatments was extracted using the DirectZol total RNA extraction kit (Zymo Research). Three 8 M SMRT cells were sequenced on a PacBio Sequel II instrument at the BYU DNA Sequencing Center. Full-length transcripts were obtained from HiFi reads using the Iso-Seq pipeline including primer removal using lima v. 2.7.1 [52], clustering using Iso-Seq3 v. 4.0.0 [52], mapping to both haplotype genome assemblies using pbmm2 v. 1.12.0 [52] and collapsing using Iso-Seq3. The transcriptome was evaluated for completeness using BUSCO as above, except in transcriptome mode. Additionally, to specifically annotate genes involved in drought, evidence-based predictions were directly inferred from a drought-response RNA-seq experiment (see below) using previously described methods [53]. In short, trimmed RNA-seq reads were aligned to each haplotype genome assembly using HISAT2 v2.2.1 [54] and used to develop five genome-guided transcriptome assemblies. Consolidated transcripts from the pooled assemblies were identified by Mikado v2.3.4 [55] using splice junctions identified by Portcullis, open reading frames identified by TransDecoder v5.5.0 [56], and homology of the transcripts to the UniProtKb Viridiplantae database release 2023_03 identified by BlastX v2.13.0 [36]. The GffRead [57] utility from Cufflinks was used to create protein and nucleotide sequences from the gene models produced by Mikado.

Gene models were predicted ab initio for each masked assembly using BRAKER3 v3.0.3 [58] in ETP mode with the Mikado transcript and protein files as inputs. The homology-based method utilized GeMoMa v1.9 [59] using reference gene annotations from *Brachypodium distachyon* (L.) P. Beauv. v3.1 [60, 61], barley (*Hordeum vulgare* L.) cv ‘Morex’ V3 [62], *L. perenne* cv ‘Kyuss’ v2.0 [19], and *L. perenne* P226 [21] reference genomes as well as the mapped RNAseq and Iso-Seq data (above) for splice site prediction. Gene models from Iso-Seq, RNA-seq, ab initio, and homology-based methods were pooled and consolidated using Mikado as described above then analyzed using cd-hit v4.8.1 [63, 64] with the sequence identity threshold of 1 to identify duplicate proteins and TEsorter v1.4.6 [65] using the REXdb Viridiplantae v3.0 database [66] to identify transposable elements. Duplicates and TEs were removed from the.gff file with AGAT v1.0.0 [67] to obtain the final gene models merged from Iso-Seq, RNA-seq, ab-initio, and homology-based methods.

The final gene models were functionally annotated separately for each haplotype using EnTAP v1.0.0 [68] configured to use the NCBI RefSeq [38] plant (release 220), NCBI BLAST nr (downloaded Oct 23, 2023), UniProtKB/Swiss-Prot (release 2023_04), and eggNOG [69] (v5.0.2) databases. To provide gene expression data for EnTAP, 63 SRA datasets with a wide variety of tissue types and treatments in *L. perenne* were downloaded with the SRA Tookit v3.0.2 and, with the trimmed RNAseq reads described below, aligned to the assemblies using Bowtie v1.3.1 [70]. Individual alignment files were merged and sorted using Samtools then used in EnTAP for expression analysis (stage 1 only using RSEM v1.3.3 [71]) to obtain a list of gene models with expression evidence. Independently, all gene models were annotated by EnTAP without gene expression filtering (skip stage 1) employing TransDecoder v5.7.1 for frame selection, DIAMOND v2.1.8 [72] for similarity searching, and eggNOG-mapper v2.1.12 [73] and InterProScan v5.64–96.0.0 [74] for gene family and ontology analysis. Only transcripts with expression evidence and/or annotation were kept using AGAT to filter and custom scripts to add the functional annotations to the.gff file. Transcriptome completeness was evaluated on the nucleotide sequences from the gene models using BUSCO in transcriptome mode as above.

Each haplotype fasta sequence was used as input for The Extensive *de novo* TE Annotator (EDTA v2.2.2) [75] with the --anno 1 and --sensitive 1 flags to annotate transposable elements. The distribution of genes, GC content, and Copia and Gypsy repeats were obtained using BEDTools in the same manner as counting telomeres described above and plotted using Circos v0.69-8 [76]. Organelle genomes were annotated using GeSeq [77] with MPI-MP chloroplast land plants and *Lolium arundinaceum*, *Lolium multiflorum*, and *Lolium perenne* NCBI RefSeqs as references. Organelle genomes were visualized using OGDRAW [78].

### Genome comparisons

To produce synteny plots between haplotypes, genomic sequences were input with the fast preset function of DEEPSPACE v0.1 [79]. Genomic structural and sequence variants were identified between haplotypes using minimap2 with the -ax asm5 preset and --eqx flag and analyzing the alignment file with Synteny and Rearrangement Identifier (SyRI) software v1.7.0 [80]. In a similar vein, syntenic blocks of predicted protein sequences from each genome were identified by using BLASTp v. 2.16.0 [36] and their genomic locations in MCScanX v1.0.0 [81], filtered to at least 15 collinear gene pairs per block. These protein sequence collinearity data were input into Synvisio [82] to produce synteny plots between the ‘Manhattan’ haplotypes and two previous *L. perenne* reference genome assemblies [19, 21]. Syntenic genes, assembly gaps, and original HiFi read mappings were visualized around the boundaries of inversions in Persephone v10.1.9180 [83] to identify spurious inversions. Assembly gaps or breaks in read coverage at inversion junctions were taken as evidence of assembly artifacts and inversions were corrected to match the reference genomes.

### LEA family proteins

Homologs of Late Embryogenesis Abundant (LEA) proteins were identified from the ‘Manhattan’ genome sequence and annotated gene models and classified according to the Pfam database. BLASTP and TBLASTN [36] were employed to identify ‘Manhattan’ LEA proteins and genes using *Arabidopsis thaliana* [9], *Zea mays* L [84]., and *Oryza sativa* L [85]. LEA protein references as queries, with e-value cutoffs of 1e^−5^. The hmmsearch function in HMMER3 [86] was also used with default parameters to independently identify ‘Manhattan’ proteins with LEA protein Pfam domains. The coordinates of the BLAST and HMMER hits were cross referenced with the perennial ryegrass genome annotation using bedtools intersect [31] to identify all potential LEA genes.

LEA protein candidates from these three sources were clustered alongside the LEA reference proteins using CLANS [87] at the MPI Bioinformatic Toolkit [88] to confirm LEA protein group relationships. The clusters were categorized based on the reference proteins found within the same cluster. BLASTP, against the non-redundant protein database of NCBI, was used to determine the most similar proteins to the two clusters that did not contain reference proteins. The hmmsearch function with the DnaJ Pfam domain (PF00226) was used to filter DHN-like proteins that had the DnaJ domain but no DHN domain. All perennial ryegrass LEA proteins were re-clustered with *A. thaliana* NHL genes retrieved from TAIR [89] to filter the portion of LEA_2 proteins (LEA_2-like) that were members of the NHL gene family cluster. Both DHN-like and LEA_2-like genes were removed from analysis.

The predicted protein sequences from the ‘Manhattan’ annotation for all LEA genes were further validated for accurate processing sites. Protein sequences were aligned within families using MAFFT [90], and bootstrap (500 iterations) supported UPGMA clustering dendrograms were generated in Geneious Prime (Auckland, New Zealand) based on the alignments. Dehydrin motifs were extracted from Battaglia et al. [13] and Szlachtowska and Rurek [91], and inputted into the Geneious Prime’s motif search to query against the DHN genes. Likewise, the three motifs predominant in LEA_1 and LEA_4 genes were extracted from Battaglia et al. [13] and Cuevas-Velazquez et al. [92] and identified in ‘Manhattan’ sequences. To identify ABA response motifs in DNA regions 2,000 bp upstream from LEA genes, the ABA response ABRE (ACGTGTC) and LTRE (GGCCGACGT) motifs [93] were searched using the fuzznuc tool from EMBOSS [94] implemented in Geneious Prime with up to two mismatches allowed for detection. To visualize the genomic locations of LEA genes, karyoploteR v1.28.0 [95] was used to plot their positions as markers on the chromosomes of both perennial ryegrass haplotypes.

### RNA-seq for expression evidence

‘Manhattan’ and three breeding lines, 2142, 2951, and 2972, all derived from the cultivar ‘Paradigm’ (NexGen Turf Research, Tangent, OR) were used for a drought imposition and RNA-seq experiment to test expression levels of LEA genes under drought conditions. These same plants were previously analyzed in a RNA-seq comparison to two other turfgrass species [16], but those data were not tied to annotated genome locations as expression counts were based on a *de novo* transcriptome assembly from the RNA-seq reads. For their growth and drought treatment, seeds of three sibling plants of each germplasm source were surface sterilized and germinated in the presence of 10 ppm Tilt (Syngenta, Wilmington, DE) to eliminate endophytes prior to transplanting. Seedlings were maintained for three months of growth in a PGR15 growth chamber (Conviron, Winnipeg, Canada), in Corvallis, OR, and then split into two clones for control and drought treatment contrasts. The split clones from each of the three sibling plants (biological replications) were grown for four more months at 50% humidity, 14 h days, and 23 °C/18°C day/night temperatures, after which one clonal replicate was subjected to drought stress while the other was kept under control growth chamber conditions. For the control conditions, the three plants from each germplasm source were watered to 80% gravimetric field capacity every other day for three weeks. For the drought treatments, the three plants from each germplasm source were watered to 27% gravimetric field capacity each day, also for three weeks. To assess treatment responses, leaf water content and turf vigor scores were recorded prior to tissue sampling for RNA extraction. Leaf water content was recorded as a ratio of dry to fresh leaf tissue weights. Turf vigor scores ranged from 1 to 9, where 1 corresponded to a dead plant and 9 to a healthy and actively growing plant [96]. Analysis of variance with entries and treatments as fixed effects, and Tukey HSD mean separations, were conducted in R [97].

Total RNA was extracted using a Direct-zol RNA extraction kit (Zymo Research, Irvin, CA) from leaf tissue of each sample after treatment, and at least 20 million Illumina PE150 reads were obtained from each sample (Omega Bio-tek, Norcross, GA). Both R1 and R2 raw Illumina reads were evaluated for quality using fastqc v0.11.5 [98] and high-quality reads were trimmed using Trimmomatic v0.39 [99] with the parameters ILLUMINACLIP: TruSeq3-PE.fa:2:30:10, LEADING:5, TRAILING:5, SLIDINGWINDOW:4:20, and MINLEN:51. Trimmed reads for each individual sample were aligned to each of the two haplotype assemblies using HISAT2 v2.2.1 [54] with default parameters, the alignment sorted by Samtools v1.17 [100], and the htseq-count utility of HTSeq v2.0.8 [101] was used in union mode with the assembly and.gff annotation file to produce feature counts. Raw counts were pre-filtered to at least 10 counts in at least 5 samples before differentially expressed genes (DEGs) were identified using DESeq2 v1.38.3 [102] with the main effect of condition (drought vs. control) in the model. Only transcripts with a corrected false discovery rate of *P* < 0.01 and a log_2_ fold change ≥ 1 were considered DEGs. Variance stabilized transformed counts were used with the plotPCA function within DESeq2 to create PCA plots, and with the EnhancedVolcano R package [103] to create volcano plots. Variance stabilized but non-transformed counts of haplotype-1-mapped genes were used to present heatmaps and to test for expression enrichment of LEAs between drought and control treatments. Enrichment was tested using genes in each family as a gene set in GSEA v4.3.3 [104] with the minimum number of genes in a set as 1 and the number of permutations as 1000 and the ranks of each LEA gene plotted within each family using a custom script in R.

## Results

### Genome assembly and annotation

Flow cytometry results indicated the genome size of ‘Manhattan’ as approximately 2.3 Gb. Five 8 M SMRT cells of whole-genome sequencing produced 5.26 million PacBio HiFi reads totaling 84 Gb (approximately 37× coverage). Analysis of k-mers from these reads provided estimates of 2.75 Gb genome size with 2.12% heterozygosity and 66.4% repetitiveness. Dovetail Genomics produced 425 million paired-end 2 × 150 Omni-C reads totaling 127 Gb. Utilizing the HiFi and Omni-C reads for assembly and scaffolding (Supplemental Figure S1) provided a haplotype-resolved assembly with two haplotypes initially with 1,506 and 895 scaffolds. The removal of 396 (13,335,369 bp) and 190 (6,911,040 bp) chloroplast, 13 (1,017,356 bp) and 8 (884,676 bp) mitochondria, 256 (9,184,422 bp) and 77 (2,592,056 bp) non-plant, and 674 (37,684,931 bp) and 492 (40,637,499 bp) repetitive scaffolds for each haplotype resulted in two haplotype assemblies with 167 and 128 filtered scaffolds, each at 2.3 Gb with N50’s near 340 Mb and the largest scaffold over 400 Mb (Table 1). Both haplotypes contain 98% of BUSCO orthologs, LAI scores over 30, and less than 50 gaps per scaffold. The seven largest scaffolds in each assembly contain 99.3% of the total length, comprise 98% of the estimated genome size, include all 98% of the BUSCO orthologs, and represent the seven haploid chromosomes of perennial ryegrass.

**Table 1:** Metrics of the ‘Manhattan’ perennial ryegrass (Lpman) assembly by haplotype

Metric	Lpman haplotype 1	Lpman haplotype 2
Assembly		
Scaffold count	167	128
Largest scaffold (bp)	413,829,865	407,779,546
Total length (bp)	2,261,789,309	2,286,290,473
GC (%)	44.30	44.36
N50 (bp)	336,284,115	341,507,219
L50	4	4
N90 (bp)	261,619,625	263,393,956
L90	7	7
Gaps per chromosome	49.4	43.0
Assembly BUSCO		
Complete	4,793 (97.9%)	4,788 (97.8%)
Complete single copy	4,536 (92.7%)	4,512 (92.2%)
Complete duplicated	257 (5.3%)	276 (5.6%)
Fragmented	10 (0.2%)	12 (0.3%)
Missing	93 (1.9%)	96 (2.0%)
Total searched (polaes_odb10)	4,896	4,896
Repeats		
Copia LTR	115,903, 113 Mb, (4.98%)	100,555, 123 Mb, (5.36%)
Gypsy LTR	361,451, 558 Mb, (24.65%)	371,893, 581 Mb, (25.42%)
Unknown LTR	740,713, 856 Mb, (37.86%)	844,066, 861 Mb, (37.68%)
LINEs	48,981, 36 Mb, (1.59%)	44,896, 32 Mb, (1.41%)
Total TIR	392,726, 201 Mb, (8.87%)	398,782, 211 Mb, (9.22%)
Helitron	241,275, 111 Mb, (4.91%)	229,055, 98 Mb, (4.3%)
Total interspersed repeats	1,935,312, 1,885 Mb, (83.35%)	2,025,740, 1,918 Mb, (83.9%)
LAI	30.16	30.09
Annotation		
No. of genes	43,347	43,803
No. of mRNA	60,948	61,754
Percent of assembly covered by genes	5.7	5.7
No. of single-exon genes	15,273	15,438
Mean gene length (bp)	2,971	2,983
Mean no. of exons per mRNA	5.6	5.7
Mean no. of introns per mRNA	4.0	4.0
No. of mRNA functionally annotated	54,459	53,663
Percent mRNA functionally annotated	89.4%	86.9%
Annotation BUSCO		
Complete	4,677 (95.5%)	4,697 (95.9%)
Complete single copy (no isoforms)	4,376 (89.4%)	4,392 (89.7%)
Complete duplicated (no isoforms)	301 (6.2%)	305 (6.2%)
Complete single copy (with isoforms)	2,935 (60.0%)	2,960 (60.5%)
Complete duplicated (with isoforms)	1,742 (35.6%)	1,737 (35.5%)
Fragmented	52 (1.1%)	42 (0.9%)
Missing	167 (3.4%)	157 (3.2%)
Total searched (polaes_odb10)	4,896	4,896

**Fig. 1:**
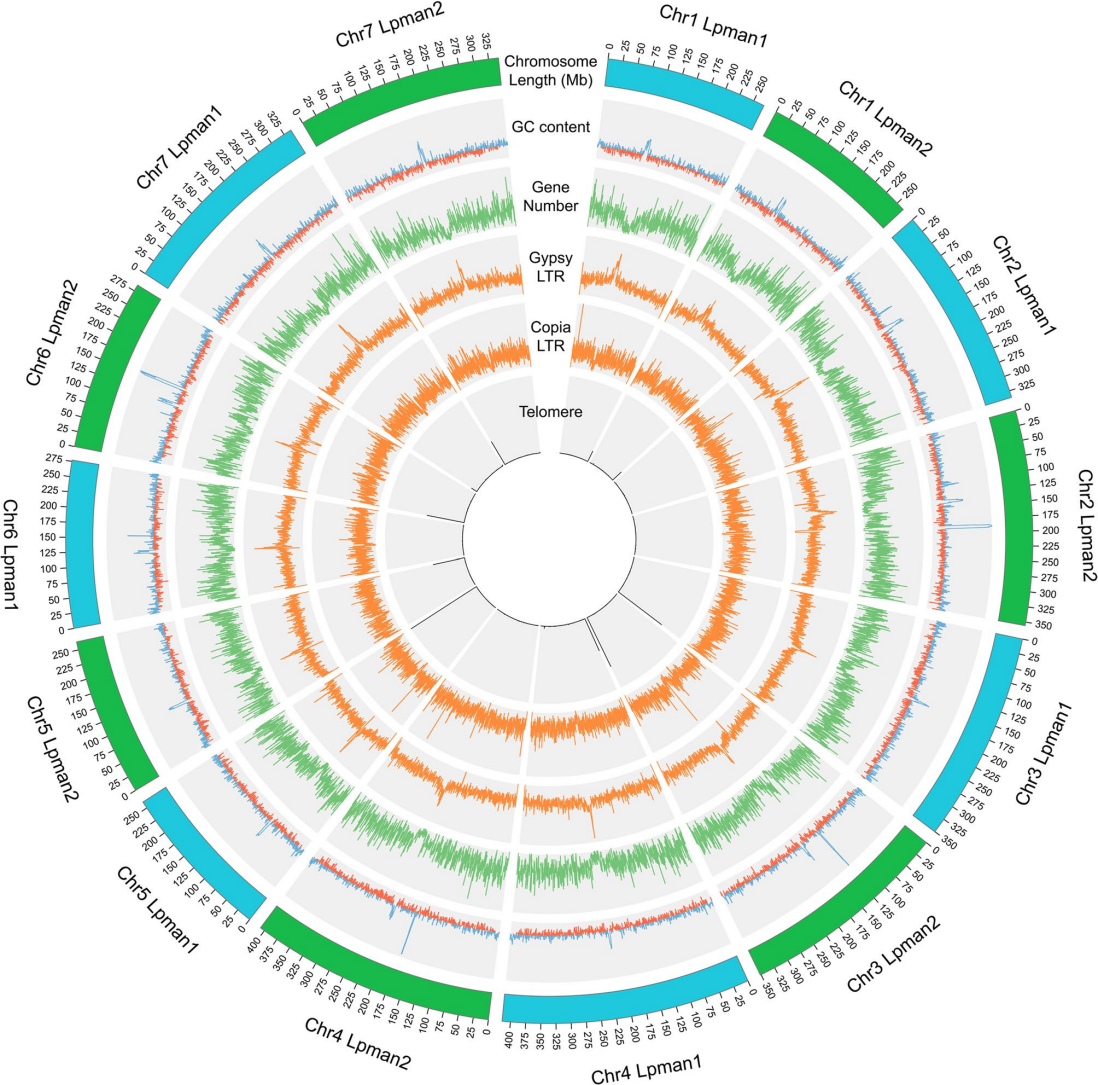
Circular representation of the ‘Manhattan’ Lpman haplotype 1 (Lpman1) and Lpman haplotype 2 (Lpman2) perennial ryegrass chromosomes. Graphs are, from outside towards center, chromosome length in Mb, distribution of GC content (percentage), gene number, Gypsy/DIRS1-like LTR repeats, Ty1/Copia LTR repeats, and telomeres in 500 Kb windows

Over 83% of both haplotypes were repetitive sequence with over 67% as LTRs, 9% as TIRs, and 4% as helitrons (Table 1). Of the retroelements over 35% were Copia or Gypsy LTR elements, which were distributed throughout the genome (Table 1; Fig. 1). Gypsy repeats were enriched near the centers (putative centromeres) of all the chromosomes and colocalized with an increase in GC content and a reduction in gene number (Fig. 1) Additional increases in GC content on Chr2, Chr3, Chr4 and Chr6 of haplotype 2 and Chr6 of haplotype 1 are locations of ribosomal and spacer sequences that were masked as repeats. Telomeric repeats were identified at the ends of chromosomes Chr1, Chr3, Chr5, and Chr7 of haplotype 1 and Chr1, Chr3, Chr5, Chr6 and Chr7 of haplotype 2 (Fig. 1).

Using PacBio HiFi reads for organelle genome assembly provided a chloroplast genome 135,268 bp in length with 409 genes, 97 tRNAs, and 16 rRNAs (Supplemental Figure S2 panel A) with GC content of 38.2%. The mitochondrial genome is 598,764 bp in length with 126 genes, 44 tRNAs, and 11 rRNAs (Supplemental Figure S2 panel B) with GC content of 44%.

### Gene annotations

Sequencing the ‘Manhattan’ transcriptome with PacBio HiFi Iso-Seq reads produced 19 Gb with 26,584 full-length transcripts and 202,192 isoforms. Since this transcriptome had 82.9% complete BUSCOs, transcripts were combined with genes models predicted from ab initio, homology-based, and direct evidence methods to annotate just over 43,000 genes with approximately 61,000 isoforms for each haplotype, respectively (Table 1). These genes spanned 129 Mb or 5.7% of the haplotype assemblies with a mean gene length of just under 3,000 bp. Despite efforts to filter gene models for transposable elements, just over 1,500 genes were functionally annotated as retrotransposons and 35% of genes were single-exon genes. The predicted transcripts had BUSCO scores of 95% complete, with 89% single-copy and 6% duplicated and over 86% were functionally annotated.

### Genome comparisons

**Fig. 2:**
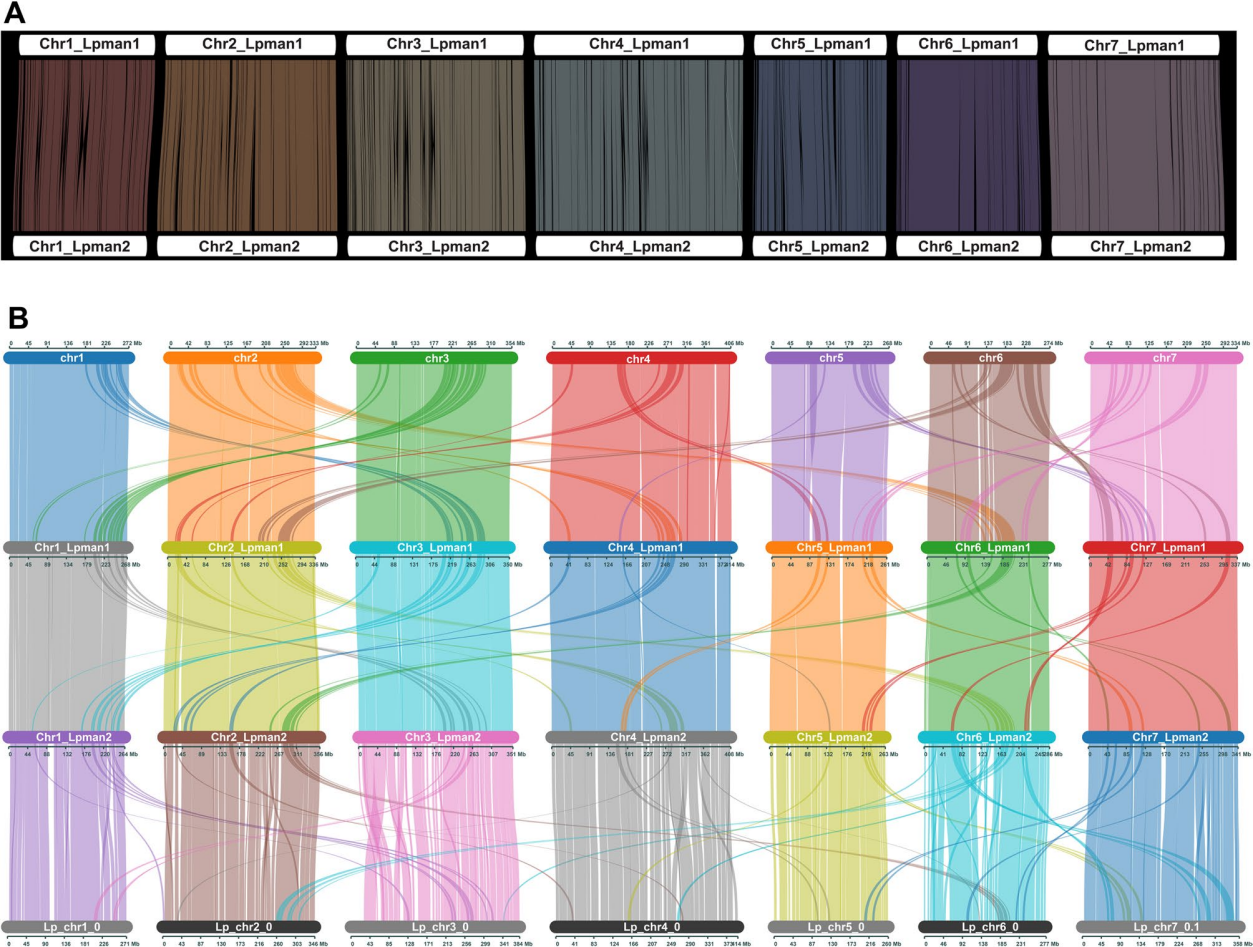
Synteny plots of the ‘Manhattan’ perennial ryegrass genome haplotypes 1 and 2. **A** Synteny based on genomic sequence. **B** Synteny based on protein sequences from annotated gene models in Lpman haplotypes 1 and 2 (middle) and previously reported reference genomes Kyuss v2 [19] (top) and P226 [21] (bottom)

Assembly metrics are similar for the two haplotype assemblies (Table 1), though haplotype 2 is 24.5 Mb longer than haplotype (1) Distribution of GC content, genes, and LTRs are also similar with the exception of increased GC content that corresponds to ribosomal genes in haplotype 2 on Chr2, Chr3, Chr4, and Chr6 (Fig. 1). All 29 major inversions between the haplotypes (Supplemental Figure S3) were visually inspected and the six that were corrected were at the end of a chromosome. Synteny maps based on genomic (Fig. 2A) or protein (Fig. 2B) sequence display overall synteny between haplotypes 1 and (2) Analysis of predicted proteins identified 95 syntenic gene blocks with 29,496 collinear gene pairs. Figure 2B and Supplemental Figure S3 illustrate the presence of intra- and inter-chromosomal duplications, 19,242 of which were identified between the two haplotypes. In addition, 185 inversions, 21,011 translocations, 3,042,731 SNPs, and 301,305 indels were identified. Genome-wide gene synteny was observed between both haplotypes and two previously published *L. perenne* genomes, Kyuss v2 [19] and P226 [21], though synteny was greater with Kyuss v2 (Fig. 2B). Interchromosomal duplicated syntenic gene blocks were present between all four assemblies in similar genomic locations.

### LEA gene family characterization

**Fig. 3:**
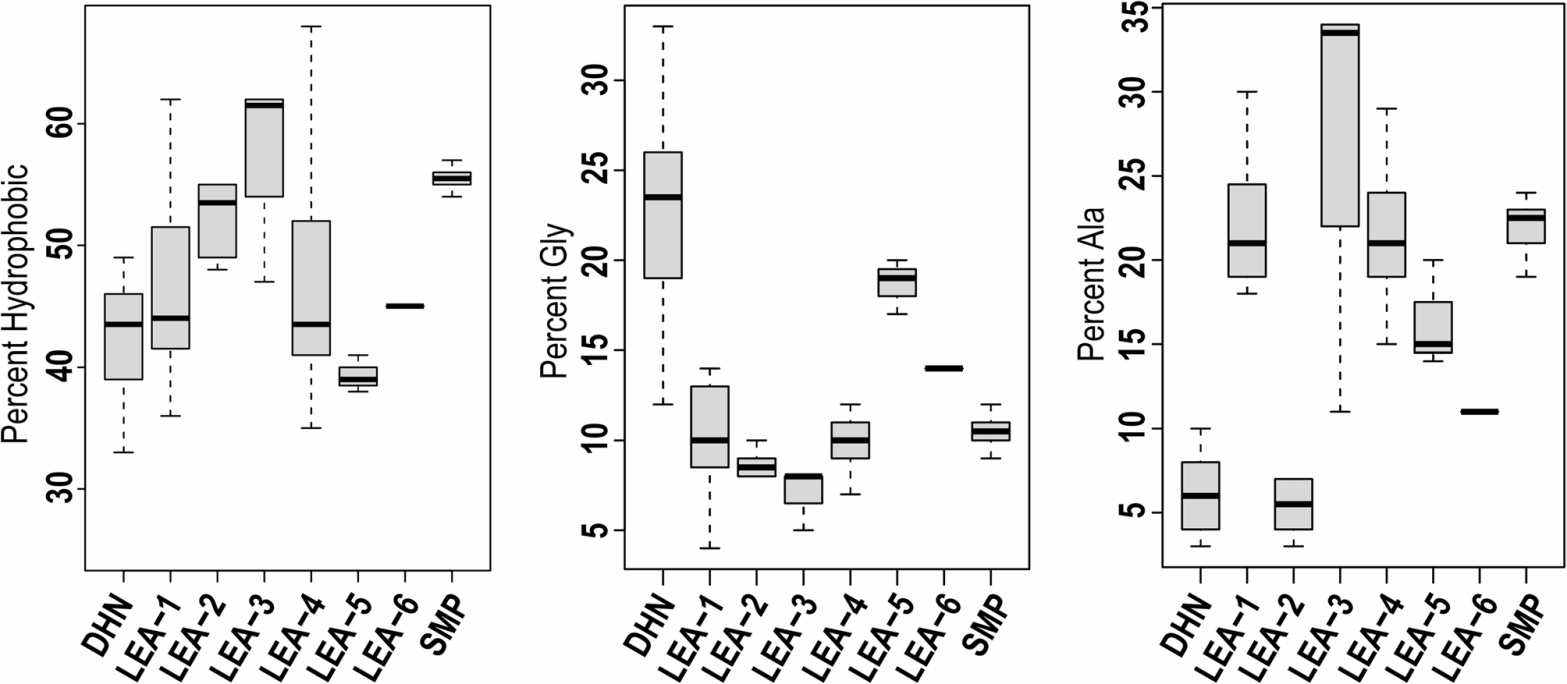
Median, quartile, and range for the total hydrophobic, percent Glycine, and percent Alanine amino acids across the eight LEA families

Using Pfam-based groupings, 72 LEA genes in 8 LEA families were found in haplotype 1 and 69 in haplotype 2 (Table 2; Supplemental Table S1; Supplemental Figure S4). The difference between the two haplotypes resulted from one less gene in each of the Seed Maturation Protein (SMP), LEA_3, and LEA_4 families in hap2. In both haplotypes, the Dehydrin (DHN) group included 14 genes and LEA_1 included 12 genes, while the LEA_2, LEA_5, and LEA_6 groups have 10 genes in total (Table 2; Supplemental Figure S4). As both haplotypes produced similar LEA numbers and profiles, we will focus on haplotype 1 characterization. Although IDPs can be characterized by hydrophobic and Glycine amino acid contents, the LEA Pfam groups showed substantial variation in both (Fig. 3). LEA_3 and SMP groups exhibited the highest percent of hydrophobic amino acids, and DHN and LEA_5 exhibited the highest percent of Glycine. Interestingly, Alanine contents were higher than Glycine for five of the eight Pfam groups (Fig. 3). Across the eight LEA gene families, all but two had three or more ABA-Response Element (ABRE) cis binding sites in their promotor regions within 2,000 bp of the start codon. Only a portion of LEA_3 and LEA_5 genes included Low Temperature Response Element (LTRE) cis binding sites.

**Table 2:** Summary of LEA genes in Lpman haplotype 1

LEA family	Pfam ID	Count	Upregulated	Downregulated	Not DEG	No Expression
LEA_1	PF03760	12	11	0	1	0
LEA_2	PF03168	6	2	0	0	4
LEA_3	PF03242	4	1	1	1	1
LEA_4	PF02987	26	8	0	6	12
LEA_5	PF00477	3	2	0	0	1
LEA_6	PF10714	1	1	0	0	0
SMP	PF04927	6	4	0	0	2
DHN	PF00257	14	6	0	2	6
Total		72	35	1	10	26

**Fig. 4:**
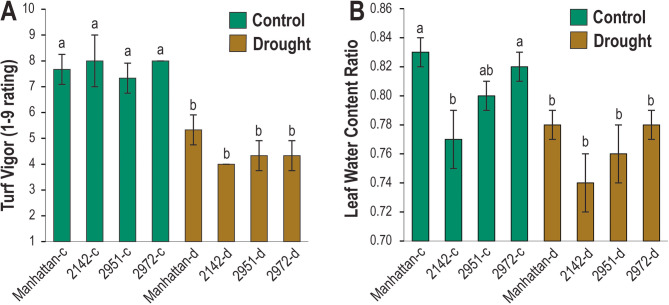
Response of four perennial ryegrass genotypes (2142, 2951, 2972, and ‘Manhattan’) under control (-c) or drought (-d) treatments. **A** Visual ratings of turf vigor on a scale from 1–9, where 1 is dead and 9 is healthy and actively growing. **B** Leaf water content as a ratio of dry to fresh leaf tissue weights. Lowercase letters indicate statistical differences at *P* < 0.05

**Fig. 5:**
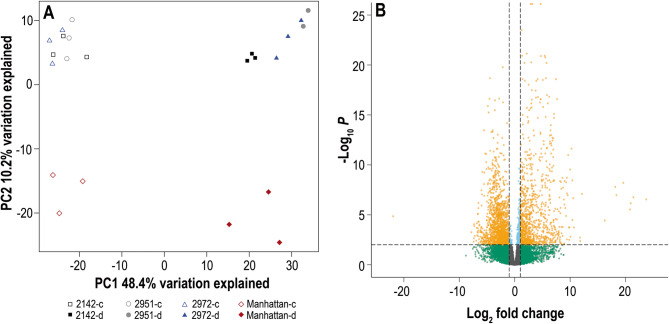
Gene expression in Lpman haplotype 1 under drought vs. control treatments. **A** Principal Component Analysis on variance stabilized transformed counts in four genotypes (2142, 2951, 2972, and ‘Manhattan’) under control (-c) or drought (-d) treatments. **B** Volcano plot of all 15,328 expressed genes. Dashed lines indicate DEG boundaries of log_2_ fold change of −1 or 1 (vertical dashed lines) and FDR *P* = 0.01 (horizontal dashed line). DEGs that are downregulated (left) or upregulated (right) are orange

**Fig. 6:**
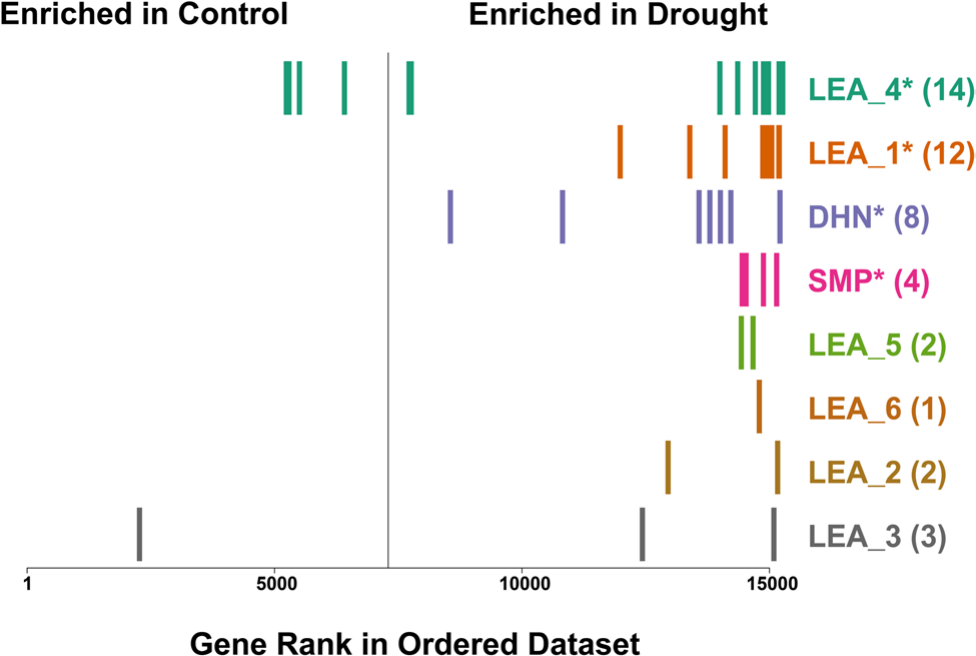
Distribution of LEA genes in Lpman haplotype 1 from enrichment analysis of drought vs. control treatments. All 15,328 expressed genes are ranked in order along the x axis from 1 (most enriched in control) to 15,328 (most enriched in drought) with the vertical line indicating the transition from control to drought. Vertical bars indicate LEA genes and are labeled by gene family on the right of the graph with the number of expressed genes in parentheses and significantly (FDR *P* < 0.05) enriched families with an asterisk

The drought treatment caused significant reductions in turf vigor across all four entries, and reductions in leaf water content in all but entry 2142 (Fig. 4). Of the ‘Manhattan’ haplotype 1 annotation gene set, 15,328 genes had expression evidence in control or drought-treated vegetative samples examined. Using the concordance of expressed genes for principal component analysis (PCA), the first axis separated drought and control treatments while the second axis separated ‘Manhattan’ from the three breeding lines (Fig. 5A). Of the expressed genes, 12,729 had functional annotations while 2,599 had no annotation or were annotated merely as uncharacterized proteins. The contrast between drought and control treatments across the four germplasm sources identified 2,495 genes that were differentially expressed with 1,029 upregulated DEGs and 1,466 downregulated DEGs (Fig. 5B). Of the 46 (64%) of the LEA genes with expression evidence, 39 were upregulated and only one LEA_3 gene was downregulated upon drought stress (Table 2). To test LEA genes for enrichment, all 15,328 genes with expression evidence were ranked based on their expression differences between control and drought treatments using the Gene Set Enrichment Analysis (GSEA) software [104]. The distribution of LEA genes within families illustrated that most LEA genes among all eight families were ranked near the extreme end that corresponded to genes induced by drought stress (Fig. 6). The four largest LEA gene families (LEA_1, DHN, LEA_4, and SMP) were significantly enriched (*P* < 0.05) while the smaller LEA families showed a trend to enrichment but did not have sufficient numbers for statistical testing. Of the 10 LEA genes near the center of the ranked graph, all but the single downregulated LEA_3 gene were not DEGs (Fig. 6; Supplemental Table S1).

**Fig. 7:**
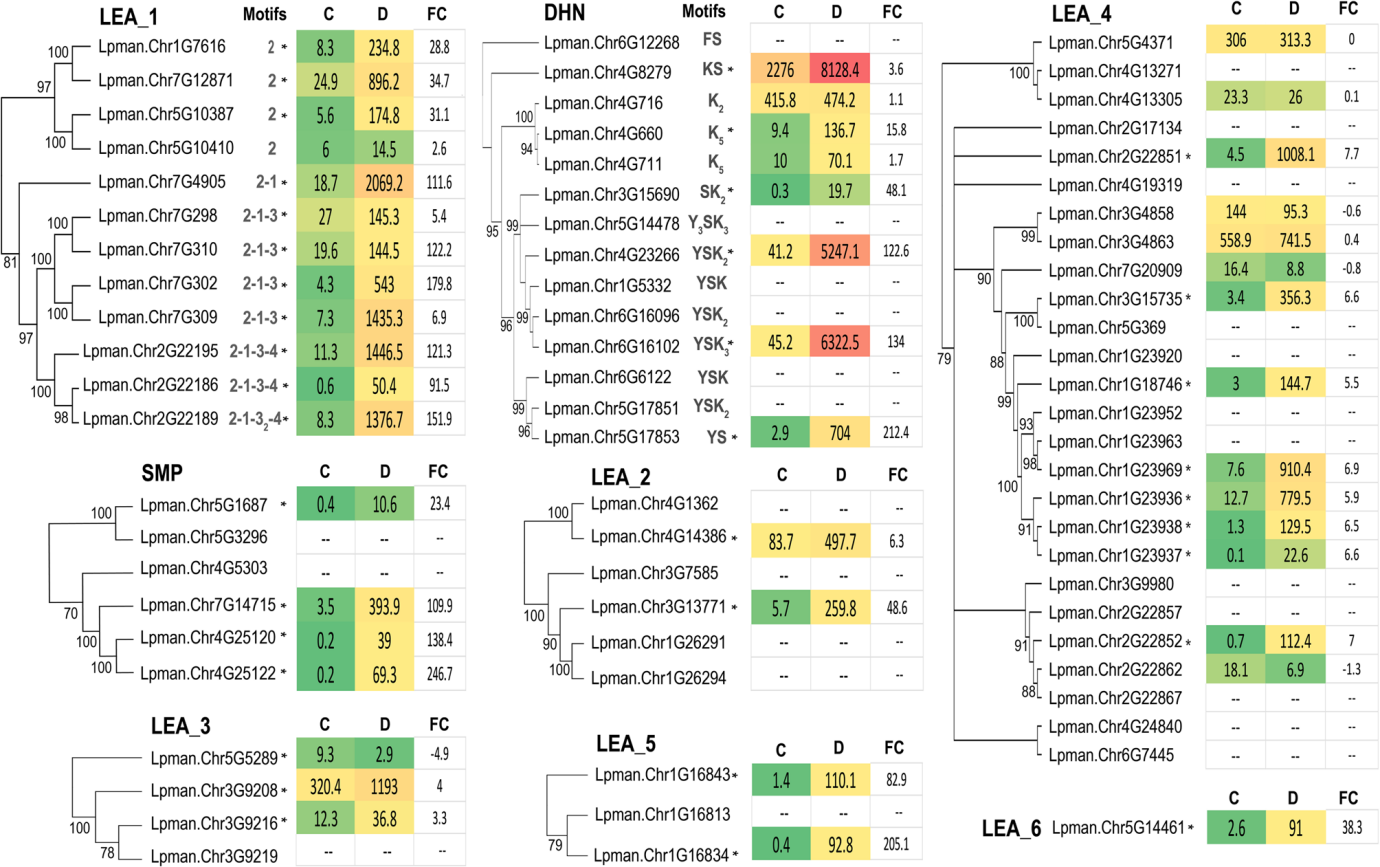
Clustering dendrograms and expression values of LEA genes in Lpman haplotype 1 with bootstrap support. Common motifs for LEA_1 and DHN proteins are shown next to each gene in gray font, and genes in all LEA families significantly induced upon drought stress are noted with an asterisk. The average normalized expression counts are reported to the right of gene names for samples under control (C) or drought (D) treatments, with the associated expression fold change (FC)

The LEA_1 Pfam group [13] included 11 genes highly induced upon drought stress in the four germplasm sources, with Lpman.Chr5G10410 showing a trend to induction that was not significant (Fig. 7; Supplementary Table S1). LEA_1 genes can be characterized by a number of possible motifs [13, 105]; and all 12 carried motif 2, eight carried motif 1, and seven carried motif 3 (Fig. 7). The LEA_1 genes separated into two subgroups of four and eight genes, respectively, with differences between them stemming predominantly in the C-terminal regions. All had low baseline expression values under control conditions while several were induced over 100-fold upon drought stress imposition. Several LEA_1 genes clustered as repeats in close proximity on chromosomes two, five, and seven (Supplemental Figure S5).

The 14 DHN genes were between 87 and 429 amino acids in length and were characterized by high Glycine content and low Alanine content (Fig. 3). The DHNs carried combinations of the three main DHN-defining motifs [13]: 12 carried at least one K-segment, 11 had a S-segment, and eight had a Y-segment. Lpman.Chr4G660, Lpman.Chr4G711, and Lpman.Chr6G102 also included omega regions reported in Malik et al. (2017) [106], while Lpman.Chr6G12268 had an F-segment in the N-terminal region rather than an S-segment [107]. Eight of the 14 DHN genes exhibited expression evidence in our drought treated vegetative tissues, and six were DEGs significantly induced upon drought stress (Fig. 7). Lpman.Chr4G23266 and Lpman.Chr6G6102 genes, having Y_n_SK_n_ motifs that are enriched in monocots, were also characterized with low expression under control conditions and over 100-fold increases in expression upon drought stress. Most of the DHN genes were in gene-repeat clusters in chromosomes four, five, and six (Supplemental Figure S5).

The LEA_4 Pfam group included 26 annotated genes, which is the largest of the eight LEA gene families in this study. Of those 26, 14 had expression evidence and 8 were DEGs induced upon drought stress (Fig. 7). A single cluster of LEA_4 genes in a tandem repeat on Chromosome 1 included four of those eight DEGs, of which Lpman.Chr1G23969 and Lpman.Chr1G23936 exhibited low expression under control conditions but were induced over 50-fold upon drought stress. Another cluster on Chromosome 2 included four genes, two of which were DEGs. Of the five smaller LEA Pfam families, all contained at least one gene with high induction upon drought stress. The single LEA_3 that was downregulated upon drought stress was also characterized by low expression counts under control conditions (Table 2; Supplementary Table S1; Supplementary Figure S3).

## Discussion

### Genome assembly

To comprehensively characterize LEA genes and evaluate their response to drought in turf-type perennial ryegrass, we developed an annotated, haplotype-resolved genome assembly for the variety ‘Manhattan’, which is called Lpman. Assembly metrics, BUSCO and LAI scores, low gap content, and telomeric repeats (Table 1; Fig. 1) indicate the haplotypes are highly complete and contiguous. The LAI scores over 30, well above the “Gold” classification [44], suggest that the repeat space is well represented in the assembly even after filtering 1,166 repetitive scaffolds totaling 78,322,430 bp in length between the two haplotypes. Comparisons to previously published perennial ryegrass genomes P226 [21] and Kyuss v2.0 [19] suggest that this assembly is also correct. As the first genome assembly of turf-type perennial ryegrass, Lpman provides a foundation for in-depth genomic analyses and tools to study the genetics that differentiate turf vs. forage-types. Instead of a pseudohaploid assembly that contains a single consensus of both haplotypes of a diploid individual with phase switching within scaffolds, haplotype-resolved assemblies contain complete sequences of both haplotypes representing the entire diploid genome [26, 108]. Haplotype-resolved assemblies allow the identification of presence/absence variation (PAV), copy number variation (CNV), and structural variation (SV) within an individual, genetic information that is missing in pseudohaploid assemblies [109]. In Lpman, the first haplotype-resolved assembly in *L. perenne*, over 40,000 structural variants and over 3,300,000 SNPs and indels were observed between haplotypes 1 and 2. Combining the two haplotypes of this assembly with P226 and Kyuss v2.0 provides valuable resources to apply pangenomics and comparative genomics to eludicate evolutionary and genome-wide variation useful for breeding improved varieties in this important forage and turf species.

The genome size of both haplotypes, which was similar to the flow cytometry estimate and the observed repetitive element content dominated by LTR retrotransposons, aligns with the forage-type perennial ryegrass genomes P226 and Kyuss v2.0, suggesting a conserved overall genomic structure across different genotypes. Since LTR Gypsy repeats include centromeric retrotransposons [110], the enrichment of these repeats illustrate the metacentric or sub-metacentric perennial ryegrass chromosomes, with the centromeres of Lpman aligning with both P226 and Kyuss v2.0. Over half the 24.5 Mb difference in length between haplotype 1 and 2 comes from the combined length of the ribosomal sequences masked as repeats on Chr2, Chr3, Chr4 and Chr6 of haplotype 2 (spikes in GC content in Fig. 1). Having both haplotype sequences may prove valuable for studying repetitive elements within the genome.

To identify a comprehensive gene set to characterize LEA genes, our annotation pipeline leveraged long-read and RNA-seq data for directive evidence, as well as ab initio and homology-based gene prediction methods. The high BUSCO scores of the predicted transcripts (96%) highlights the completeness of the annotation. The number of genes identified in both haplotypes (43,347 and 43,803) was fewer than in P226 (54,629 v3 high confidence genes) but more than Kyuss v2.0 (38,765), which is reflected in gene densities of 5.7%, 5.7%, 7.0%, and 5.2%. respectively. These gene densities are consistent with other large plant genomes characterized by extensive repetitive elements. The high proportion of single-exon genes and the presence of functionally annotated retrotransposons within the gene set highlight the challenges inherent in annotating repeat-rich genomes and suggest further investigation and manual curation to improve the annotation in future versions. Further investigation is also required to understand the role of repetitive elements and ancestral genome duplication events to explain the presence of interchromosomal duplicated syntenic gene blocks across all three assemblies (Fig. 2B).

### LEA analysis

The number of LEA genes identified in this genome assembly, 72, was comparable to the 51 found in Arabidopsis [9], the 112 in cereal rye (*Secale cereal* L.) [111], 99 in barley (*Hordeum vulgare* L.) [112], and 67 in *Brachypodium distachyon* [113]. Members of all eight Pfam LEA families were detected, with the three largest corresponding to those detected and characterized most effectively in initial LEA discovery efforts [8, 9]. LEA genes were found on every chromosome and often occurred in clustered groups of repeats within families (Supplementary Figure S3) [111, 114]. The percentage of their hydrophobic residues ranged from 32 to 70%, with LEA_2, LEA_3, and SMP families having more hydrophobic amino acids than the other families (Fig. 3). This trend is similar to Arabidopsis LEA gene families, where LEA_2 and LEA_3 families were considered atypical and exhibited higher relative hydrophobicity [9].

Of the 72 LEA genes identified in the Lpman haplotype 1 annotation dataset, 46 were expressed in these vegetative tissues, and 39 were differentially expressed upon drought. The 54% proportion of LEAs induced upon drought stress in vegetative tissues is consistent with the varied tissue and stress responses found for LEA genes [9]. The LEA family was among the most enriched gene groups induced upon drought stress. Moreover, nearly all LEA DEGs had very low basal expression under control conditions. This expression profile makes them ideal for transformation per se, but also ideal for the use of their ABRE-rich promoter regions in other vector constructs. The DHN Lpman.Chr4G8279 gene (Fig. 7; Supplementary Table S1), one of the few with high basal expression, would provide an alternative expression profile that is highly expressed in control conditions but still induced strongly upon drought stress. With specific LEA genes responding to drought stress in different plant lineages [12, 106, 115, 116], those specifically induced upon drought in perennial ryegrass can provide candidate genes for genetic approaches to improving drought tolerance.

LEA_1 genes were some of the first described in other plants, have shown strong induction upon water deficit stress [105], and have been transformed and shown to improve water deficit stress [117]. However, these genes have also shown a lack of consistent functional changes upon different protein conformations [10]. In this study the LEA_1 gene family was the only family where all genes showed expression evidence, and all but one were DEGs induced upon drought stress (Fig. 7; Supplementary Table S1). The two clades within this LEA_1 family are consistent with other species, but unlike dicot groupings [13] these clades were not distinguishable by peptide length. Rather, one clade contained four genes of varying length with imperfect versions of the main motif 2 while the remaining eight genes of varying length included imperfect versions of three other motifs reported in dicot species [105]. Seven of the 12 LEA_1 genes occurred in two clusters, one cluster of three genes on chromosome 2 and another of four genes on chromosome 7. Each of the two clusters could be defined by motif structures and each included genes induced over 50-fold by drought stress (Fig. 7).

Dehydrins are perhaps the most well-characterized family of LEA genes, and the 14 identified in the Lpman haplotype 1 annotation dataset is similar in number found in other monocots [14, 106, 114, 118]. The DHN genes in perennial ryegrass had the highest percentage of Glycine and the lowest percentage of Alanine among the LEA families, also similar to reports in other plants [91, 119]. Three clusters of DHN repeats were found on chromosomes 4–6, similar to findings in Triticeae species [114]. For each group of genes, a predominant expressed DEG existed alongside related genes with little or no expression evidence (Supplementary Figure S3). The main Y, S, and K motifs in DHNs can have substantial variation [106], but none have been examined in cool season perennial grasses. As with other monocots, the Y_n_SK_n_ motifs were the predominant motif in perennial ryegrass and no Y_n_K_m_ motif carrying genes were detected [12, 107]. Unlike other monocots [106], however, the perennial ryegrass DHNs included three K_n_ genes with expression evidence in vegetative tissues and transcript induction upon drought stress. Only two of the seven Y_n_SK_n_ DHN genes had expression evidence, which may result from tissue or treatment specific roles [14]; yet both were highly induced upon drought stress. As the two Y_n_SK_n_ DEGs in this study, Lpman.Chr6G16102 and Lpman.Chr4G23266, had little expression under control conditions but over 50-fold increases upon drought conditions, they would be candidates for further genetic studies in perennial ryegrass.

LEA_2 was previously reported as the most numerous LEA family member across several plant species [12], but LEA_4 was the largest in *Arabidopis* [9] and in this Lpman haplotype 1 annotation dataset. One possibility for the differences in LEA_2 abundance might be the inclusion of LEA_2-like genes that share some motifs with NHL (NDR1/HIN1-like) genes [120]. Some of those LEA_2-like/NHL genes are also expressed during seed maturation and can respond to ABA, but are characterized more for their responses to biotic stresses [12, 120].

As with other LEA families, the LEA_4 family can also be characterized with motifs such as the AYDKA—AKD motif reported in Arabidopsis [13, 121]. However, the LEA_4 family members generally lack high sequence similarity [9] and in this study only the largest clade within the LEA_4 gene family carried imperfect repeats of that motif. Six of eight genes on chromosome 1, and in a clade with 99% bootstrap support, were DEGs, and four of those six were part of a repeat cluster (Fig. 7; Supplementary Table S1; Supplementary Figure S3). Interestingly, of the few studies that reported LEA genes in Poaceae, an LEA_4 (group 3) gene obtained from heterologous primers from an Italian ryegrass cold-stimulated EST database [122], was found to have SNPs associated with leaf water content in a diverse perennial ryegrass population [5]. The primers from that previous study matched Lpman.Chr2G22857, which is one of the LEA_4 genes in that repeat cluster on chromosome 2 (Supplementary Figure S3); but did not show expression evidence in this study (Fig. 7).

The other five LEA families each comprised fewer than six genes and have relatively sparse information available for characterization. An LEA_3 gene on chromosome 5 was the sole LEA with reduced transcript levels upon drought stress, but had low basal expression as well that may preclude biological effects (Supplementary Table S1). Although the low numbers of genes in each of the five smaller LEA gene families precluded enrichment tests, all included at least one DEG induced upon drought stress in vegetative tissues (Supplementary Table S1). With drought stress an increasing challenge for turfgrasses in the western USA, LEA gene characterization can help direct selection efforts.

## Conclusions

This study provides a high-quality haplotype-resolved assembly developed from the turf-type perennial ryegrass cultivar ‘Manhattan’ and a comprehensive characterization of its LEA gene families. The assembly provides valuable genomic resources for the identification of genes and understanding the genome of turf-type perennial ryegrass to assist in developing improved varieties, as exemplified by the identification and characterization of LEA genes and their response to drought conditions. LEA genes from each of the eight Pfam groups were spread throughout the genome, often in clusters, though not all genes in a cluster were expressed under drought stress in vegetative tissues. LEA genes from all groups were induced upon drought stress with specific DHN gene families the most highly induced. Though the number and structure of LEA genes identified in Lpman are similar to other plants, LEA genes in perennial ryegrass are structurally diverse with unique motifs in genes expressed under drought.

## Supplementary Information

Supplementary Material 1.

Supplementary Material 2.

Supplementary Material 3.

Supplementary Material 4.

Supplementary Material 5.

Supplementary Material 6.

## Data Availability

The sequencing data supporting this project are available at NCBI (www.ncbi.nlm.nih.gov) SRA under BioProject accession PRJNA1247987. The PacBio Hifi data are accessions SRX28312808-SRX28312812, the Dovetail OmniC proximity ligation data is accession SRX28312813, the PacBio Iso-Seq data are accessions SRX28312814-SRX28312816, and the RNA-seq data are accessions SRX28289459-SRX28289482. The assembled nuclear genomes are available at NCBI as BioProject accessions PRJNA1247931 (haplotype 1) and PRJNA1247930 (haplotype 2). The annotated chloroplast and mitochondrial genomes are also available at NCBI. All the nuclear and organelle genomes and gene and TE annotation files are available on the USDA Ag Data Commons with DOI 10.15482/USDA.ADC/28791824 (10.15482/USDA.ADC/28791824).

## Abbreviations

DEG: Differentially expressed gene
DHN: Dehydrin (Pfam PF00257)
EST: Expressed sequence tag
FDR: False discovery rate
GSEA: Gene set enrichment analysis
IDP: Intrinsically disordered proteins
LAI: LTR assembly index
LEA: Late embryogenesis abundant
Lpman: *Lolium perenne* cultivar ‘Manhattan’
LTR: Long terminal repeat
TIR: Terminal inverted repeat
NHL: NHL repeat (Pfam PF01436)
SMP: Seed maturation protein (Pfam PF04927)
SNP: Single nucleotide polymorphism
