# Supplementary figures and images for: Haplotype-resolved genome assembly of ‘Manhattan’ perennial ryegrass (Lolium perenne L.) and characterization of drought responsive late embryogenesis abundant genes
Source: BMC Genomics. 2025 Nov 20;26:1125. doi: 10.1186/s12864-025-12144-1 (PMC12751607; doi:10.1186/s12864-025-12144-1)

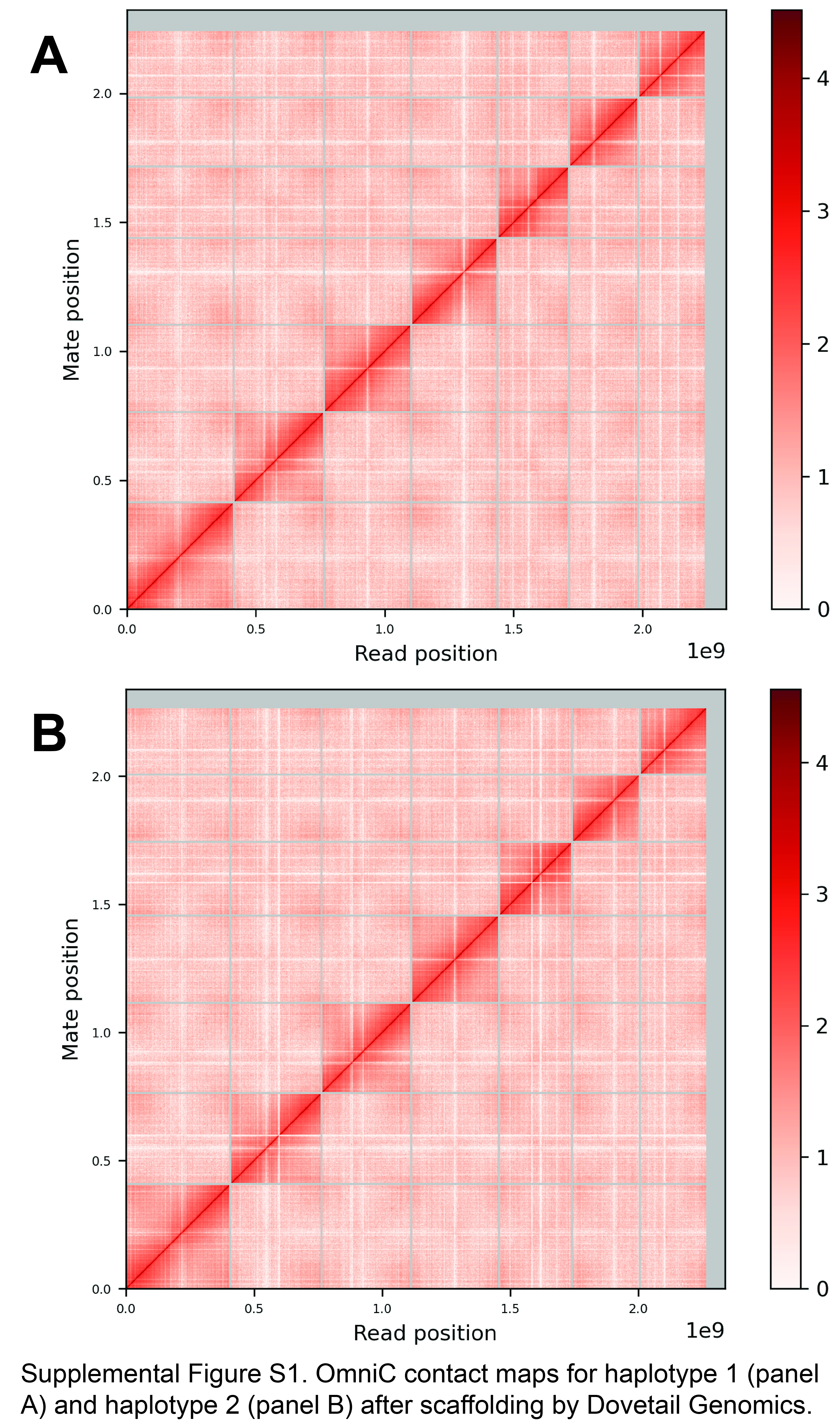

Supplement: Supplementary file 1 — Supplementary Material 1. [file 12864_2025_12144_MOESM1_ESM.jpg]

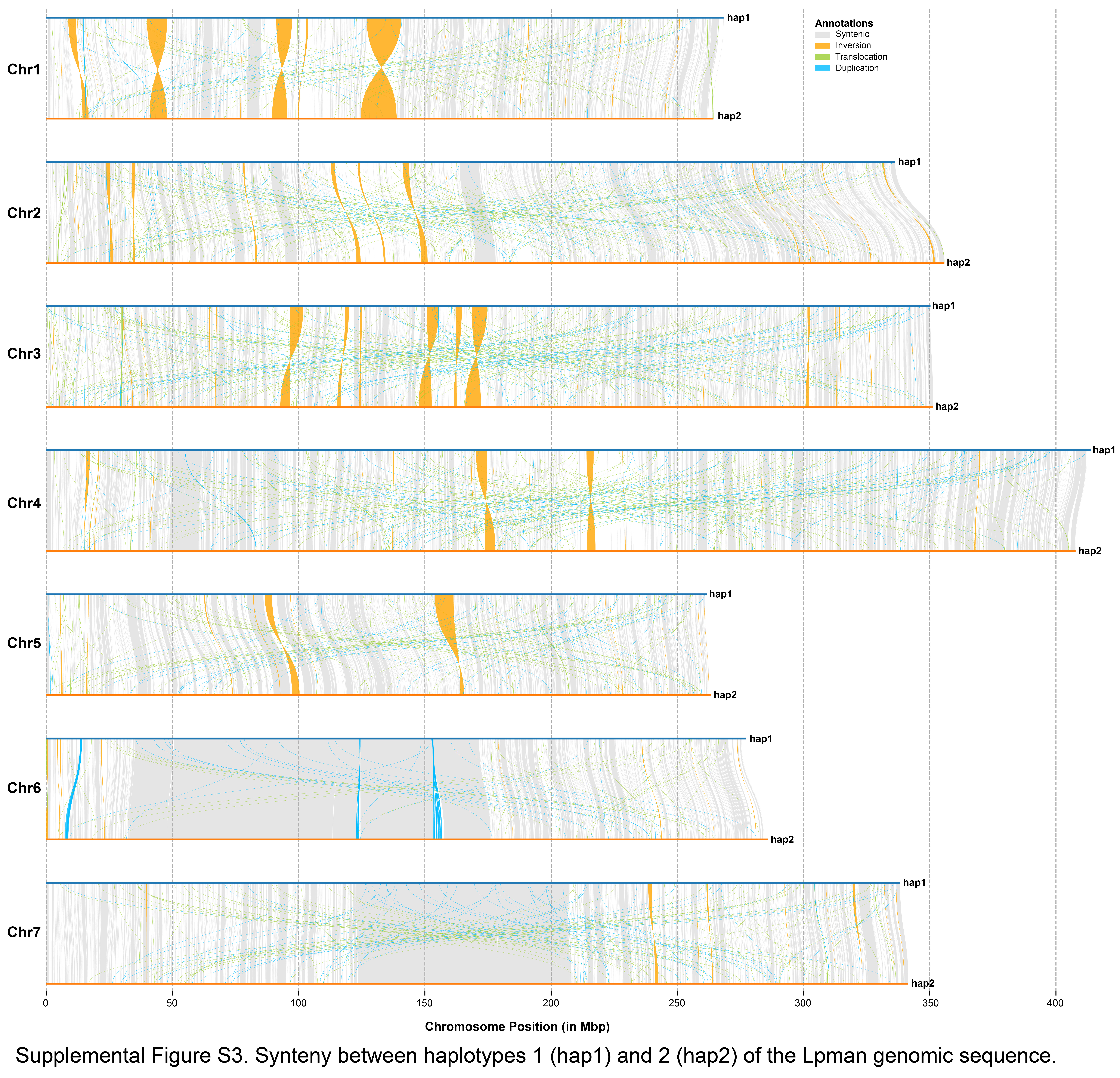

Supplement: Supplementary file 3 — Supplementary Material 3. [file 12864_2025_12144_MOESM3_ESM.jpg]

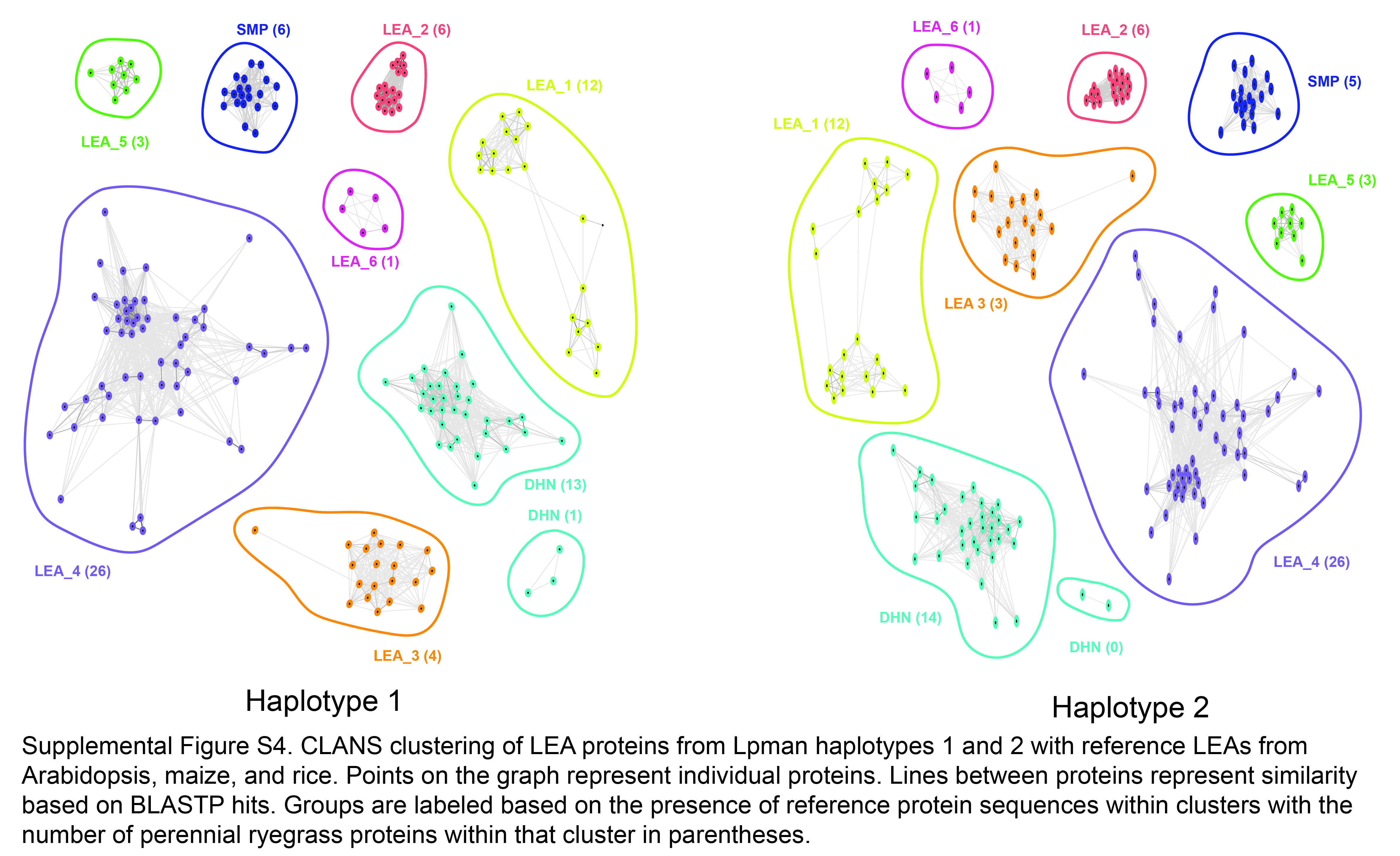

Supplement: Supplementary file 4 — Supplementary Material 4. [file 12864_2025_12144_MOESM4_ESM.jpg]

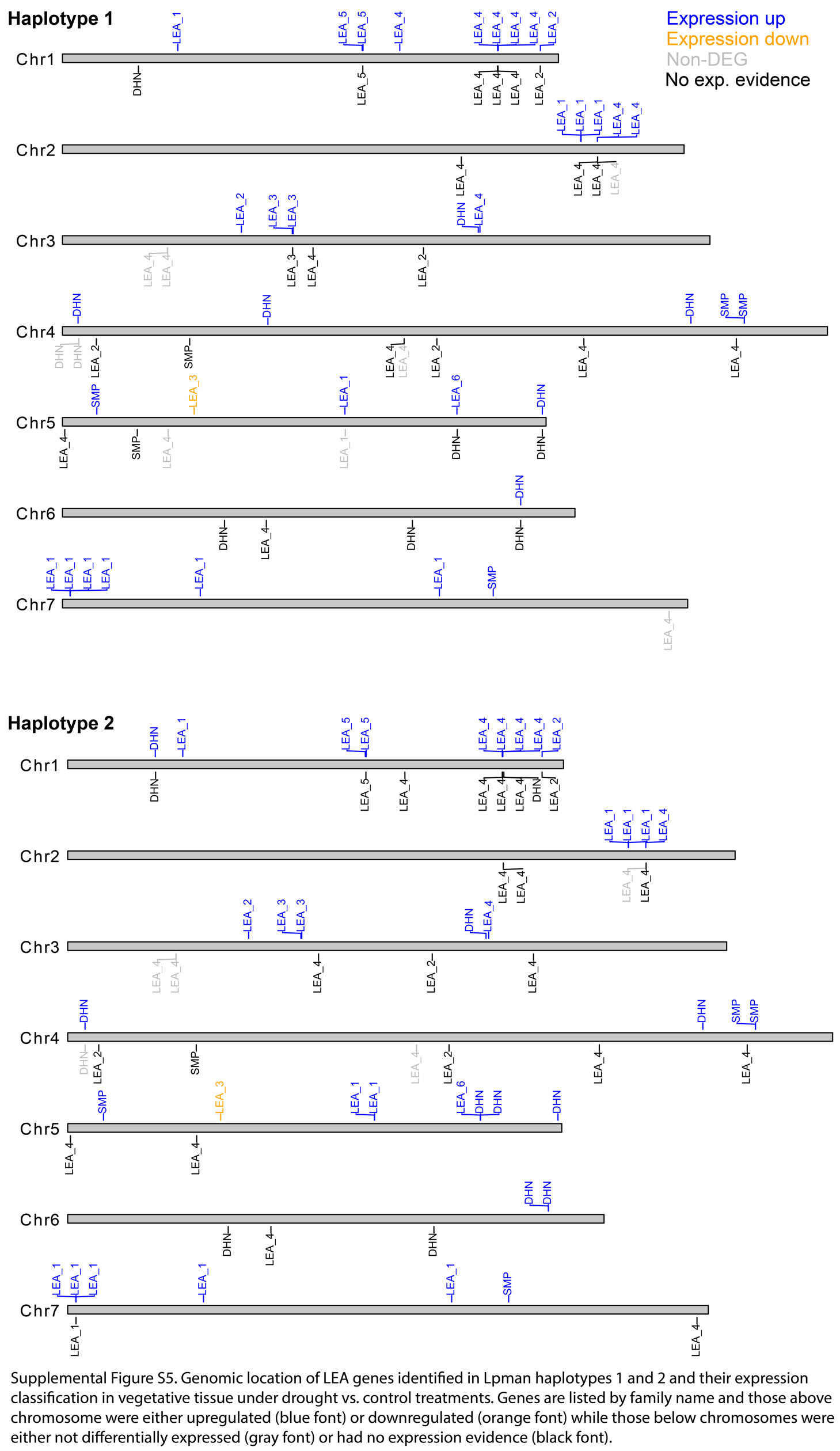

Supplement: Supplementary file 5 — Supplementary Material 5. [file 12864_2025_12144_MOESM5_ESM.jpg]
